# Design of Novel PLA/OMMT Films with Improved Gas Barrier and Mechanical Properties by Intercalating OMMT Interlayer with High Gas Barrier Polymers

**DOI:** 10.3390/polym13223962

**Published:** 2021-11-16

**Authors:** Abdul Shakoor Shar, Caili Zhang, Xieqing Song, Yunxuan Weng, Qiuyue Du

**Affiliations:** 1 College of Chemistry and Materials Engineering, Beijing Technology and Business University, Beijing 100048, China; ashakoorshar1234@outlook.com; 2 Fenghua Research Institute of Ningbo University of Technology, Ningbo 315500, China; songxieqing110@163.com; 3 School of Artificial Intelligence, Beijing Technology and Business University, Beijing 100048, China; duqiuyue@btbu.edu.cn

**Keywords:** poly(lactic acid), OMMT, gas barrier films, nanocomposites

## Abstract

Polymer/clay composites are an innovative class of materials. In this study, we present a facile method for the preparation of biodegradable and robust PLA/organomodified montmorillonite (OMMT) composite films with excellent gas barrier performance. When the design of PLA/OMMT composite films, in addition to making OMMT have good intercalation effect in the matrix, the compatibility of intercalating polymer and matrix should also be considered. In this work, two polymers with high gas barrier properties, namely poly(vinyl alcohol) (PVA) and ethylene vinyl alcohol copolymer (EVOH), were selected to intercalate OMMT. The morphology and microstructures of the prepared PLA/PVA/OMMT and PLA/EVOH/OMMT composites were characterized by the X-ray diffraction measurement, scanning electron microscopy, and differential scanning calorimetry. It was shown that the good dispersibility of PVA in the PLA matrix, rather than the intercalation effect, was responsible for the improved gas barrier and mechanical properties of PLA/PVA/OMMT composite. The elongation at break increases from 4.5% to 22.7% when 1 wt % PVA is added to PLA/OMMT. Moreover, gas barrier of PLA/PVA1/OMMT measured as O_2_ permeability is 52.8% higher than that of neat PLA. This work provides a route to intercalate OMMT interlayer with high gas barrier polymers and thus can be a useful reference to fabricate PLA/OMMT composites with improved gas barrier and mechanical properties. A comparison of oxygen permeabilities with existing commercial packaging films indicates that the biodegradable PLA/PVA/OMMT may serve as a viable substitute for packaging film applications.

## 1. Introduction

The shelf life of food is very important for maintaining its freshness and quality [1]. Studies have shown that increased food wastage due to food deterioration results in decreased food safety, which ultimately leads to increased malnutrition and hunger [2]. The most effective way to prevent food spoilage is to develop advanced food packaging materials [3]. When it comes to packaging, plastics are the most widely used material owing to low cost, convenient usage, and good availability. Along with the other petroleum-based materials, plastics represent one of the principal environmental pollutants particularly affecting the marine ecosystem. Plastics are used in different industries, where the highest share of global plastics consumption goes to packaging industry [4]. Although highly recyclable, only 14% of plastics are collected globally for recycling, whereas only 5% are converted into high-quality products [5]. Since the beginning of massive usage of plastics, many research efforts have been invested in the development of alternative materials as a substitution for synthetic polymers. Ideally, these alternatives should be polymers from natural and renewable sources with good biodegradability. Various natural polymers have been used as functional, biodegradable packing materials due to their nontoxicity, biocompatibility, and environmental friendliness [6]. Therefore, the current study represents a continuation of the work on the development of natural and biodegradable polymers for food packaging.

Polylactic acid (PLA) is one of the most promising alternatives to petroleum-based polymers in the food packaging industry, owing to its excellent mechanical robustness [7], renewability, nontoxicity, and good gas barrier properties [8]. Other beneficial PLA properties that make PLA such an attractive green food packaging material include bioavailability [9], low energy input for the preparation, biodegradability [10], and low cost of raw material [11]. Moreover, PLA exhibits excellent thermal stability, with the onset of degradation temperature between 330 and 350 °C [12], mechanical strength expressed as high Young’s modulus of ca.3 GPa, tensile strength of ca. 50–70 MPa, elongation at break of around 4%, and impact strength of 2.5 KJ/m^2^ [13]. As PLA might be produced from agricultural products such as potato, cane molasses, cassava, starch, sugar beet, and corn, etc. [14], it represents an ecofriendly thermoplastic material [8].

Montmorillonite (MMT) is one of the most widely used nanoclay. MMT behaves as a smectic liquid crystal, which means that it forms oriented planes that stack and slide one over another in the crystal. This property further allows significant expansion of interlayer space or swelling. When combined in various polymer–MMT composites, it enhances the performances of pure polymers by increasing the interfacial area between the polymer matrix and clay platelets due to the exfoliation of crystalline clay bundles.

Considering food packaging applications, enhanced mechanical properties [15], decreased gas permeability [16], and increased hydrophobicity of polymer–MMT composites increase the research interest in this field [17]. Due to its specific structure, unique intercalation properties, high strength, stiffness, and low cost, clay minerals are gaining more attention as a functional ingredient for different polymers. In recent years, organic modified MMT (OMMT) has been applied extensively in combination with natural polymers such as PLA [18]. Studies had provided evidence that the gas barrier and mechanical [19] and thermal properties of clay–polymer nanocomposites are significantly improved upon the introduction of even a small amount of clay (0.5 wt %) [20]. Recently, polyvinyl alcohol (PVA)/MMT nanocomposites have been fabricated, and the material exhibited excellent gas barrier properties, high transparency, and low sensitivity to humidity [21]. In another study, polyethylene/nanoclay composites were prepared and applied for food packaging due to good gas barrier properties [22]. Ko et al. [23] produced mechanically enhanced PLA/PBAT nano-filled composite films by melt blending, using HNTs and OMMTs as the fillers. The obtained films exhibited an improvement in rheological properties and mechanical strength.

**Scheme 1:**
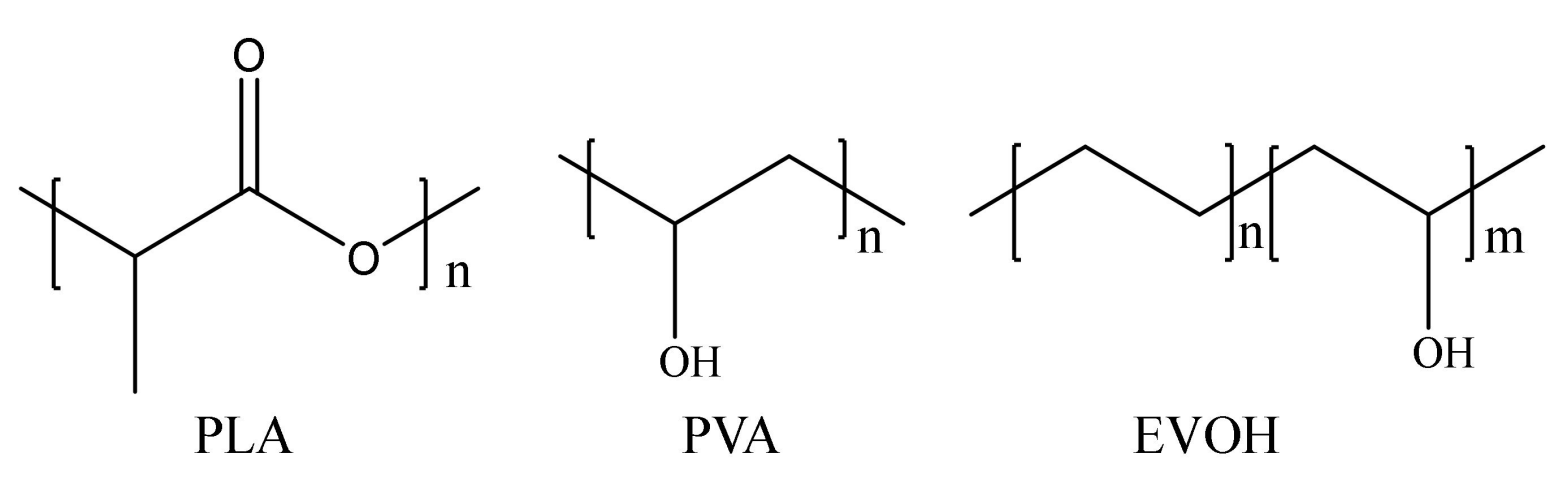
The chemical structure of PLA, PVA, and EVOH.

In this work, two polymers with high gas barrier properties, namely PVA and ethylene vinyl alcohol copolymer (EVOH), were intercalated between OMMT layers to obtain robust and biodegradable composite films. The effect of PVA and EVOH addition on the intercalation and dispersibility of OMMT in PLA along with mechanical and gas barrier properties of PLA/OMMT composites were studied in detail. The chemical structures of PLA, PVA and EVOH are shown in Scheme 1.

## 2. Experimental

### 2.1. Materials

The PLA (4032D, Mn = 106,000 g/mol, Mw = 223,000 g/mol) with approx. 2% _D_-LA was supplied by NatureWorks (Minnetonka, MN, USA). The OMMT (I.24 TL), with a length/thickness ratio of 80 where the organic modified is 18-amino stearic acid, was purchased from Nanocor Inc. (Schaumburg, IL, USA). Before processing, OMMT and PLA were oven-dried at 80 °C for 12 h. PVA (17–88) with a degree of polymerization 1700, and alcohol content of 88% was obtained from Aladdin. Ethylene vinyl alcohol copolymer (EVOH) resins supplied by Kuraray Co., Ltd., Tokyo, Japan were H171 grade and contain 38 mol% ethylene with the density of 1.17 g cm^−3^ and a melt flow index of 1.8 g/10 min at 190 °C. PVA and EVOH granules were oven-dried at 80 °C for 12 h before internal mixing with PLA and OMMT.

### 2.2. Composite Films Preparation

The composite plates were prepared using the internal mixer (Haake Rheometer) operating at 180 °C for 5 min with a 60 rpm rotation speed. Next, the composite material was granulated using a crusher. After that, it was molded using a hydraulic press operating at 10 MPa and 180 °C to produce 80–90 µm thick films. The composition of different nanocomposite films prepared in this work is listed in Table 1.

### 2.3. Characterization

The X-ray diffraction (XRD) patterns of all samples were recorded on a Bruker D8 Advance X-ray diffractometer (Germany), which uses a cooper radiation source operating at 40 kV and 40 mA. The scan speed was set to 0.5°/min between 2° and 10° and 2°/min from 10° to 40°. The scanning electron microscope (SEM) analysis of the freeze-fractured composite samples was done on the Phenom Pro machine (Netherlands) operating at 10 kV. Differential scanning calorimeter (DSC) experiments were conducted on a TA Q20 instrument to determine the thermal properties of fresh polymer pellets. The sample was heated in an inert atmosphere obtained by passing the nitrogen gas at 20 mL/min flow rate to 190 °C at a heating rate of 20 °C/min. This temperature was maintained for 5 min, and then the sample was cooled to 30 °C; this temperature was kept for 3 min, and the final heating run proceeded with 10 °C/min rate until reaching 190 °C. The integration of melting peaks provided the melting enthalpies, which were then used for the calculation of the degree of crystallinity (*χ*_c_) according to Equation (1).

(1) $$x_{c}=\frac{\Delta H_{m}}{w\%\Delta H_{m}^{0}}\times 100\%$$

where $\Delta H_{m}$ stands for melting enthalpy (J/g) of a composite; ΔHm0 is the melting enthalpy of pure, crystalline PLA (93.7 J/g) [24]; and *w* is the weight percentage of PLA in the composites.

The gas permeation properties of PLA/OMMT nanocomposite films were analyzed following ASTM D3985 standard [25]. The oxygen transmission through 10 cm^2^ film was measured using an oxygen transmission rate tester 31M (Labthink, Jinan, China) at 23 °C and 30% humidity. The reported values represent the average of three independent measurements. The tensile strength (TS) and the elongation at break (EB) of the composites was analyzed according to GB/T 1040 standard, applying a tensile rate of 5 mm/min via electric tensile tester (Shenzhen Labsans Material Testing Co., Ltd., Shenzhen, China). The final data are the average of 10–15 specimens for each sample.

## 3. Results and Discussion

### 3.1. Film Structure and Morphology

**Figure 1:**
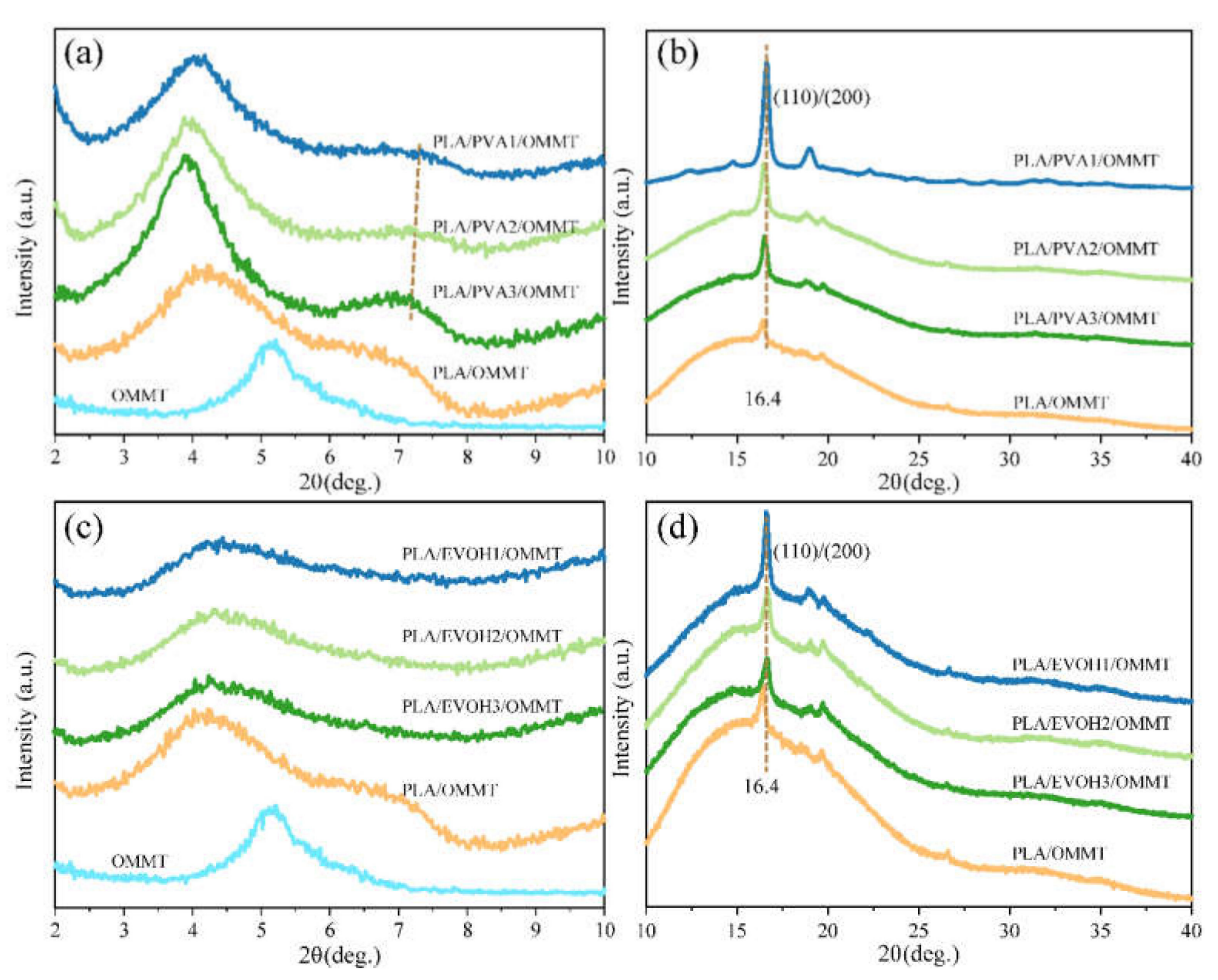
XRD curves of (**a**,**b**) PLA/PVA/OMMT; (**c**,**d**) PLA/EVOH/OMMT composite films.

The changes in the intercalation, dispersion of OMMT, and crystal structure of PLA upon the addition of PVA and EVOH to PLA/OMMT system were studied by XRD. Previously, we found that PLA/OMMT nanocomposites with 6 wt % OMMT show the highest dispersibility and the best gas barrier performance [16], so in this study, we fixed the amount of OMMT to 6 wt %. Figure 1 shows XRD curves of PLA/OMMT, PLA/PVA/OMMT, and PLA/EVOH/OMMT composites with various amounts of PVA and EVOH within 2–40°. The 2*θ* values between 2–10° correspond to OMMT in the nanocomposites, whereas neat PLA and the PLA in the nanocomposites exhibit XRD peaks between 10–40°. The observed 2*θ* values and the interlayer *d*-spacing calculated from Bragg’s law are listed in Table 2.

In this work, the structure of MMT was modified with the addition of polar, long-chain 18-amino stearic acid (HOOC(CH_2_)_17_NH_3_^+^Cl^−^), which adsorbs on the internal surface of interlayer space. In general, the enhancement in the barrier properties of polymer/OMMT composite films is mainly dependent on the dispersion level of OMMT lamella in the polymer matrix. Two factors affect the dispersion of OMMT in the PLA matrix, namely: (1) the fabrication method used for the preparation of polymer/OMMT composites and (2) the interactions between the OMMT surface and the polymer chains. The melt mixing method used in this work was considered to be a better method than the solution mixing technique because the dispersion of OMMT sheets is favored under strong shear stress [26]. Further, melt mixing does not use any solvent, which makes it a green and economical method for the large-scale production of polymer/OMMT composites.

According to our previous work [16], pure OMMT (I.24 TL) shows the primary diffraction peak at 2*θ* = 5.22° assigned to (001) plane, giving the interlayer spacing of 1.69 nm. The most intense peak of neat PLA is observed at 2*θ* = 16.4° and assigned to the diffractions from (110)/(200) planes. As shown in Figure 1, the addition of PVA and EVOH affects OMMT interlayer spacing and PLA crystallinity in PLA/OMMT nanocomposites.

The intense (001) peak typical of OMMT is observed in Figure 1a, implying that the OMMT is intercalated rather than exfoliated [27]. The gradual increase in PVA concentration resulted in a higher intensity of OMMT peak and increased *d*-spacing. All PLA/PVA/OMMT samples show higher interlayer spacing than PLA/OMMT, which proves that the addition of PVA improves the intercalation effect of OMMT in PLA. Interestingly, a new diffraction peak at 2*θ* = 6.98° appeared in the XRD spectrum of the sample with 3 wt % PVA, which is explained by the stacking of a portion of layers leading to their aggregation [28]. Figure 1b shows that there are no diffraction peaks of crystalline PVA after it has been added into the PLA/OMMT system, indicating the amorphous nature of PVA in the composites. The results in Figure 1c suggest that the addition of EVOH increases the *d*-spacing of OMMT layers similarly to PVA. On the other hand, there was no peak at 2*θ* = 6.98° in PLA/EVOH/OMMT samples, indicating weaker stacking interactions between the layers and better intercalation effect of EVOH on OMMT compared with PVA [28]. Somewhat lower interlayer spacing in the PLA/EVOH/OMMT samples compared to PVA analogs is mainly attributed to the differences in the conformations of polymer chain and interactions between the polymer chain and OMMT lamella. The relative strength of the interactions between the polymers and OMMT could be estimated using the polar solubility parameters (PSP) [29], where closer PSP values indicate stronger interactions between the molecules. The PSP values for PLA, PVA, and EVOH are 20.2, 27.7, and 25.9 J^1/2^ cm^3/2^, respectively [30,31,32], and PSP of the surfactant (18-amino stearic acid) used in OMMT is 25.1 J^1/2^ cm^3/2^ [33]. The smaller difference between the PSP of EVOH and OMMT surfactant (0.8 J^1/2^ cm^3/2^) compared with PVA–OMMT pair (2.6 J^1/2^ cm^3/2^) indicates stronger interactions of OMMT and EVOH polymer.

**Figure 2:**
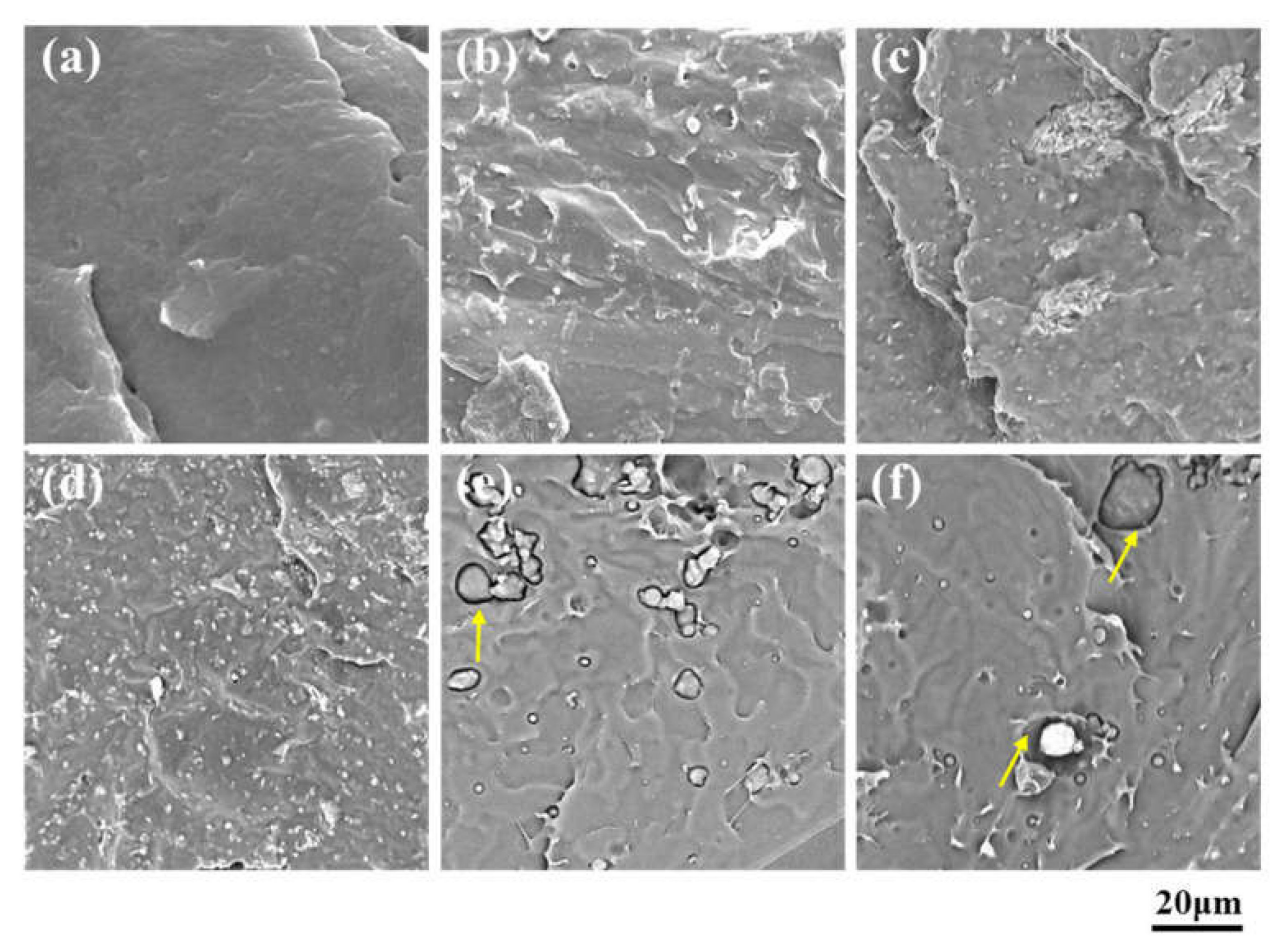
SEM images for the fractured surfaces of (**a**) neat PLA, (**b**) PLA/OMMT, (**c**) PLA/PVA1/OMMT, (**d**) PLA/PVA3/OMMT, (**e**) PLA/EVOH1/OMMT, and (**f**) PLA/EVOH3/OMMT.

**Scheme 2:**
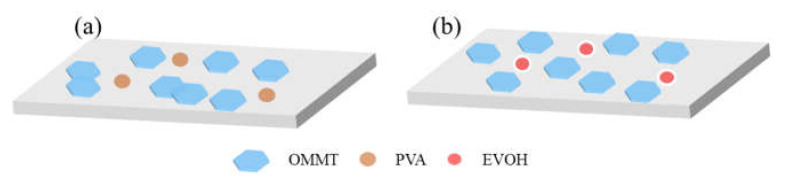
Intercalation and compatibility effect of (**a**) PLA/PVA/OMMT and (**b**) PLA/EVOH/OMMT composites.

In addition to the degree of OMMT intercalation, the gas barrier performance of PLA/PVA/OMMT and PLA/EVOH/OMMT is also related to the compatibility of intercalated polymer (PVA and EVOH) with the PLA matrix. Poor compatibility leads to structure defects at the interface or the agglomeration of intercalated polymer. The dispersion of PVA or EVOH in the PLA matrix was studied by SEM. Smooth surfaces observed for PLA and PLA/OMMT fractured films (Figure 2a,b) suggest that the OMMT is uniformly dispersed in the PLA without any aggregation. The distribution of spherical particles in PLA/PVA/OMMT and PLA/EVOH/OMMT nanocomposites given in Figure 2c–f indicates better particle dispersion of nanocomposites with PVA as a functional ingredient. SEM images also show that the increased amounts of PVA and EVOH favor the agglomeration process where a narrow gap formed surrounds the agglomerating particles (marked with yellow arrow), resulting in poor mechanical properties and poor gas barrier performance of composite films. The intercalation and compatibility effect of PLA/PVA/OMMT and PLA/EVOH/OMMT composites was illustrated in Scheme 2.

### 3.2. Crystallinity

**Figure 3:**
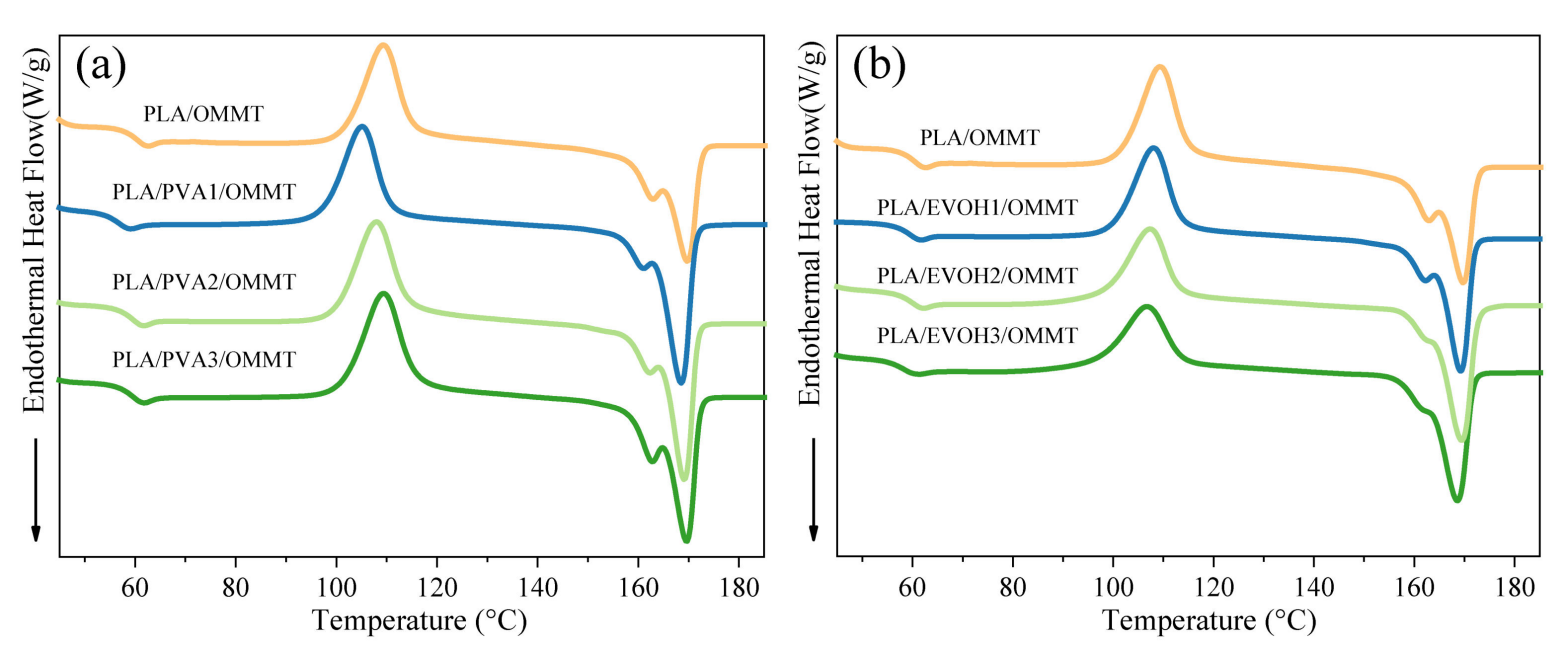
DSC curves for the second heating scan of (**a**) PLA/PVA/OMMT composites and (**b**) PLA/EVOH/OMMT composites.

The DSC curves suggest that the *T*_g_ of PLA in the PLA/PVA/OMMT and PLA/EVOH/OMMT composites were lower than those of neat PLA (Figure 3 and Table 3). This implies higher flexibility of PLA chains in PLA/PVA/OMMT and PLA/EVOH/OMMT as a result of the presence of PVA and EVOH flexible chains. Moreover, the temperature of cold crystallization (*T*_cc_) of PLA were somewhat lower in nanocomposites than in neat PLA, as PVA and EVOH chains could serve as nucleation centers to promote crystallization and reduce *T*_cc_.

Figure 3a shows that the increased content of PVA in PLA/OMMT system gradually increases *T*_cc_ and also influences the crystallinity (*χ*_c_) of PLA matrix. The addition of 1 wt % PVA increases the crystallinity of the matrix from 3.1% to 7.0% and decreases *T*_cc_ by 4.1 °C. In contrast, further addition of PVA reduces *χ*_c_ from 7.0% to 1.7% and shifts *T*_cc_ toward higher temperatures. These results suggest that there is a certain amount of PVA for optimum cold crystallization effect, while the excessive PVA content reduces the flexibility of PLA chains. This further results in the reduced compatibility between two phases and the decreased extent of heterogeneous nucleation. Figure 2b shows the changes in *T*_cc_ and *χ*_c_ of PLA matrix upon the addition of EVOH. The same trends were observed for PVA, so there is also an optimum level of EVOH for heterogeneous crystallization, above which EVOH agglomerates and hinders the movement of PLA chains.

### 3.3. Mechanical Properties

**Figure 4:**
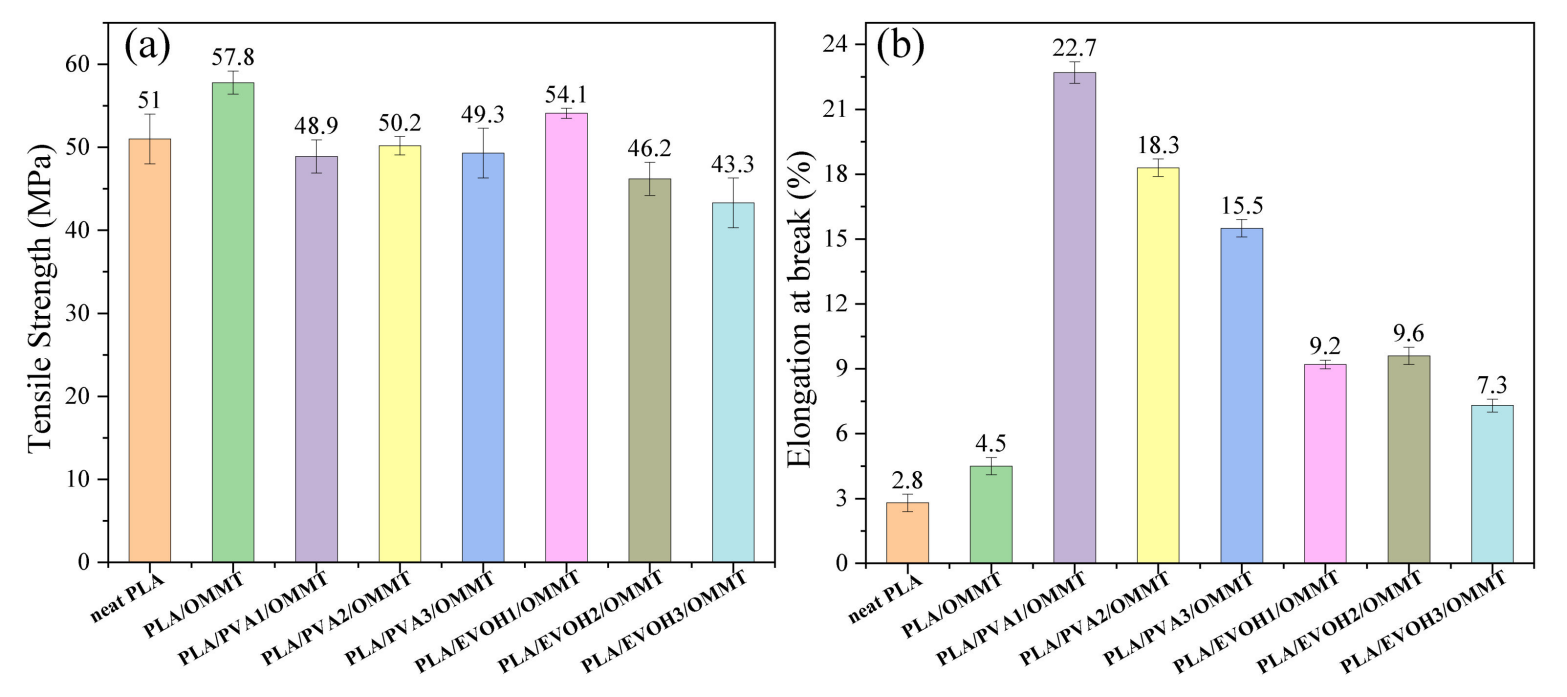
(**a**) Tensile strength and (**b**) elongation at break of PLA/PVA/OMMT and PLA/EVOH/OMMT composites.

Figure 4 shows the differences in tensile strength (TS) and elongation at break (EB) between neat PLA and PLA/PVA/OMMT and PLA/EVOH/OMMT nanocomposites. The highest TS is observed for PLA/OMMT, whereas the addition of PVA or EVOH reduced this property. In contrast, the EB of all composites with PVA or EVOH was significantly enhanced compared with PLA/OMMT (EB 4.5%), especially for PLA/PVA1/OMMT sample with an EB of 22.7%. These results might be explained by the formation of a flexible interfacial layer and good dispersibility of PVA, resulting in a toughening effect on PLA matrix. Although EVOH also exhibits a good OMMT intercalation effect, a part of EVOH that did not occupy interfacial space of PLA agglomerates in the matrix and significantly reduces the mechanical properties of PLA/EVOH/OMMT composites.

### 3.4. Gas Barrier Properties

**Figure 5:**
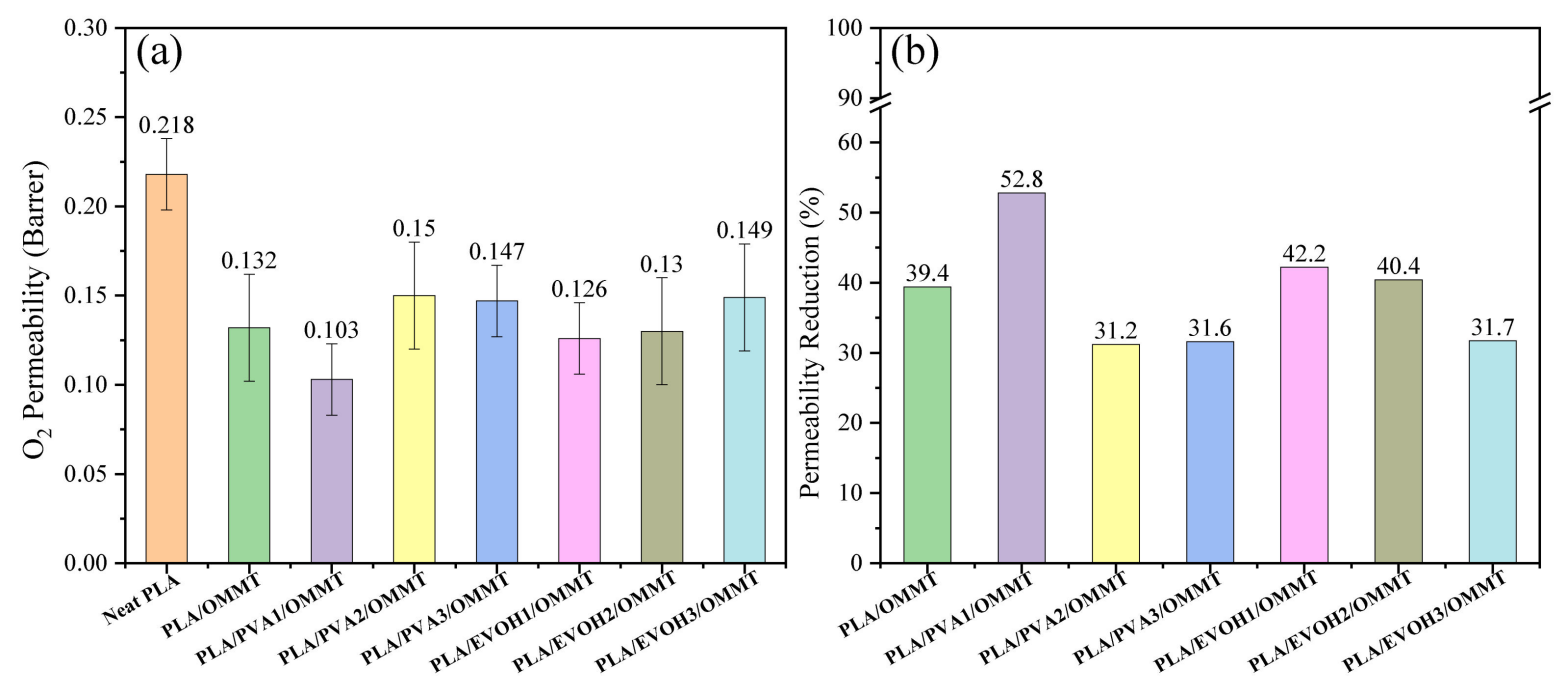
(**a**) O_2_ permeability and (**b**) permeability reduction relative to neat PLA.

The gas barrier properties of composite films were characterized by the measurement of oxygen permeability (*P*_O2_). The results are listed in Table 4 and plotted as a histogram in Figure 5 for better visualization of the differences in gas barrier performance of neat PLA and composites. Upon addition of 1 wt % PVA, the oxygen permeability reduced by 52.8% compared with neat PLA, from 0.218 to 0.103 Barrer. However, composite membranes were more permeable for O_2_ when the amount of PVA exceeded 1%. Poor dispersion of PVA in PLA matrix previously observed at higher PVA levels results in structural defects of the polymer film. When the amount of intercalated polymer is low, it occupies interstitial space and spreads homogeneously all over the matrix, which enhances the gas barrier properties of composites. Higher amount of PVA decreases compatibility between PVA and PLA matrix, leading to agglomeration, crystal structure defects, and other issues that reduce gas barrier properties.

**Figure 6:**
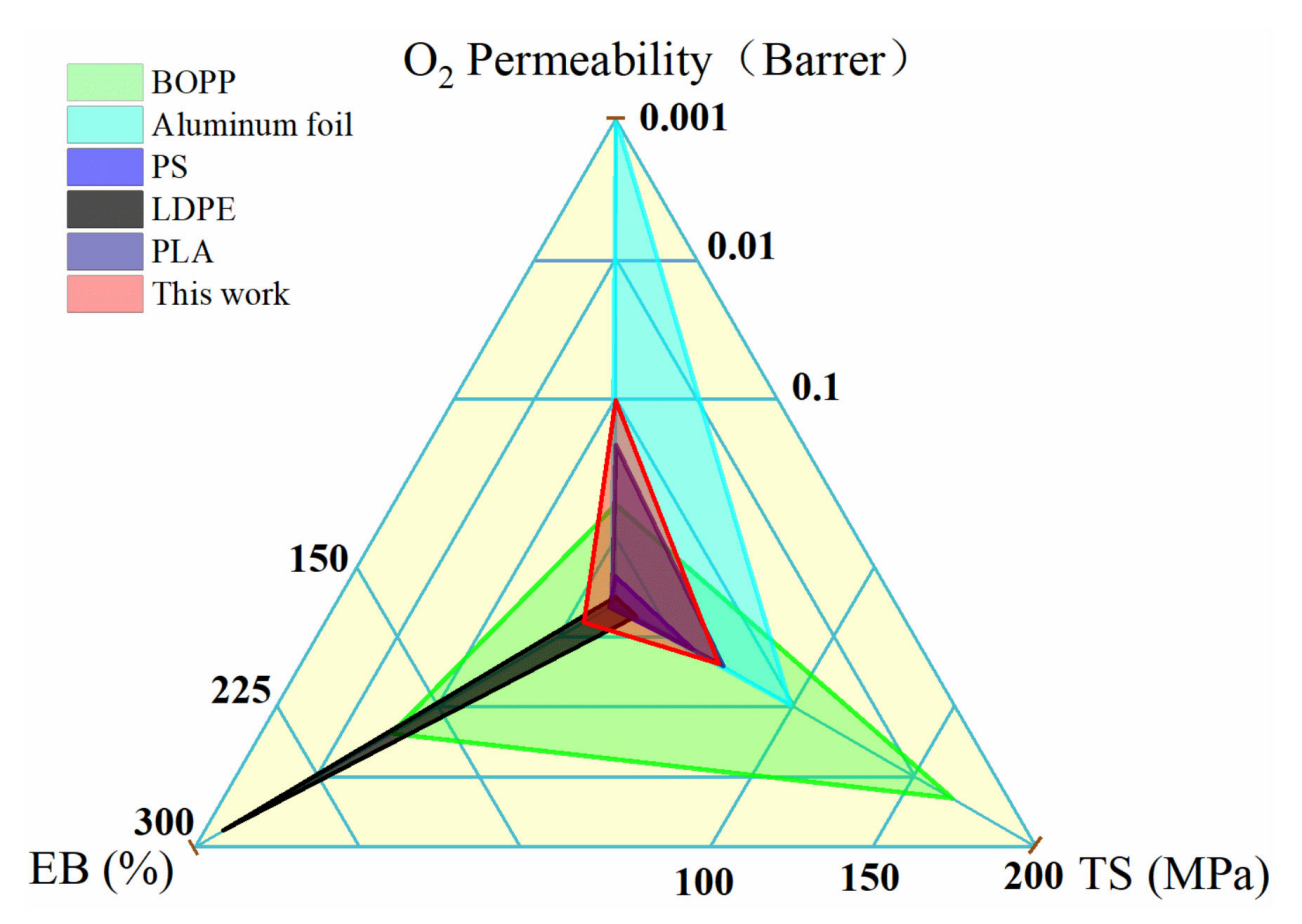
Performance triangle diagram to compare the O_2_ permeability, TS, and EB of PLA/PVA1/OMMT film with other packaging films. The overall performance of each material is represented by the area of corresponding triangle. The properties of commercial packaging films are taken from the literature: BOPP [34], aluminum foil [35], PS [36,37], LDPE [38,39], and neat PLA [16].

Next, we compared the mechanical and gas barrier properties of PLA/PVA1/OMMT film with several commercial packaging materials. The results presented in Figure 6 indicate the better overall performance of newly developed composite than some commercial materials. More specifically, the oxygen barrier performance of HDPE and BOPP is inferior to that of PLA/PVA1/OMMT film, although these two materials have superior ductility. Ductility is an important property of packaging materials, and the PLA/PVA/OMMT nanocomposite material exhibits higher ductility compared with PLA, PS, and aluminum foil. Besides, the new material is robust and shows high gas barrier properties. Most importantly, full biodegradability of PLA/PVA/OMMT composite films makes it a promising candidate for an environmentally friendly substitute of conventional, petroleum-based polymers.

## 4. Conclusions

In this work, we demonstrated a facile procedure for the preparation of PLA/OMMT nanocomposite films with excellent gas barrier properties and good mechanical robustness. It was shown that the design of composite films should consider the structure of OMMT lamella and intercalating polymer as well as compatibility between intercalating polymer and polymer matrix. Although the intercalation effect of PVA was not as good as that of EVOH, PVA had excellent dispersion in PLA matrix that resulted in better mechanical and gas barrier properties of PLA/PVA1/OMMT compared to PLA/EVOH1/OMMT composite film. The EB increased from 4.5% for PLA/OMMT to 22.7% for PLA/PVA1/OMMT, whereas oxygen permeability was decreased by 52.8% when comparing PLA/PVA1/OMMT with neat PLA film. Therefore, this work provides a route to intercalate OMMT interlayer with high gas barrier polymers and, thus, can be a useful reference to fabricate PLA/OMMT composites with improved gas barrier and mechanical properties. A comparison of oxygen permeabilities with existing commercial packaging films indicates that the biodegradable PLA/PVA/OMMT may serve as a viable substitute for packaging film applications.

## Figures and Tables

**Table 1:** The weight percentages of different compounds used for the preparation of PLA/OMMT films.

Sample Codes	PLA (wt %)	OMMT (wt %)	PVA (wt %)	EVOH (wt %)
Pristine PLA	100	0	/	/
PLA/OMMT	94	6	/	/
PLA/PVA1/OMMT	93	6	1	/
PLA/PVA2/OMMT	92	6	2	/
PLA/PVA3/OMMT	91	6	3	/
PLA/EVOH1/OMMT	93	6	/	1
PLA/EVOH2/OMMT	92	6	/	2
PLA/EVOH3/OMMT	91	6	/	3

**Table 2:** The 2*θ* values and corresponding *d*-spacing of PLA/PVA/OMMT and PLA/EVOH/OMMT composite films.

Samples	2*θ* 2~10°	*d*-Spacing (nm)		2*θ* 2~10°	*d*-Spacing (nm)
OMMT	5.22	1.69	PLA/OMMT	4.22	2.09
PLA/PVA1/OMMT	4.18	2.11	PLA/EVOH1/OMMT	4.44	1.99
PLA/PVA2/OMMT	3.88	2.27	PLA/EVOH2/OMMT	4.30	2.06
PLA/PVA3/OMMT	3.88, 6.98	2.27, 1.27	PLA/EVOH3/OMMT	4.02	2.20

**Table 3:** Thermal properties and crystallinity of the PLA/PVA/OMMT and PLA/EVOH/OMMT composites obtained from the DSC curves.

Samples	*T*_g_ (°C)	*T*_cc_ (°C)	*T*_m1_ (°C)	*T*_m2_ (°C)	Δ*H*_cc_ (J/g)	Δ*H*_m_ (J/g)	*χ*_c_ (%)
Neat PLA	58.9	112.8	163.1	170.8	23.7	24.8	1.2
PLA/OMMT	58.7	109.2	162.8	169.7	24.5	27.2	3.1
PLA/PVA1/OMMT	56.8	105.1	161.7	168.1	24.2	30.3	7.0
PLA/PVA2/OMMT	56.3	107.5	162.7	169.0	25.9	29.4	3.4
PLA/PVA3/OMMT	58.3	109.3	162.8	169.7	26.4	27.3	1.7
PLA/EVOH1/OMMT	57.3	108.2	162.3	169.4	24.3	30.7	7.3
PLA/EVOH2/OMMT	58.1	107.3	162.0	169.6	24.7	30.5	6.6
PLA/EVOH3/OMMT	57.9	106.5	162.2	168.7	25.3	30.3	5.7

**Table 4:** The results of oxygen permeability and permeability reduction of the PLA/PVA/OMMT and PLA/EVOH/OMMT composites.

Samples	OMMT (wt %)	PVA (wt %)	EVOH (wt %)	O_2_ Permeability		Change (%)
				Barrer *	10^−14^ cm^3^ cm/cm^2^ s Pa	
Neat PLA	0	0	0	0.218	1.635	0
PLA/OMMT	6	0	0	0.132	0.990	39.4
PLA/PVA1/OMMT	6	1	/	0.103	0.773	52.8
PLA/PVA2/OMMT	6	2	/	0.150	1.125	31.2
PLA/PVA3/OMMT	6	3	/	0.147	1.103	31.6
PLA/EVOH1/OMMT	6	/	1	0.126	0.945	42.2
PLA/EVOH2/OMMT	6	/	2	0.130	0.975	40.4
PLA/EVOH3/OMMT	6	/	3	0.149	1.118	31.7

* 1 Barrer = 1 × 10^−10^ cm^3^ cm/cm^2^ s cmHg = 7.5 × 10^−18^ m^2^/s.Pa = 7.5 × 10^−14^ cm^3^ cm/cm^2^ s Pa; 1 Pa = 7.5005 × 10^−4^ cmHg.

## Data Availability

The data presented in this study are available on request from the corresponding author.
